# When similarity is less disruptive: feature-sharing distractors in visual working memory under diffuse attention

**DOI:** 10.3389/fcogn.2026.1900438

**Published:** 2026-08-12

**Authors:** Jinhua Fu, Dexiang Zhang, Hehong Wu, Yaxi Li

**Affiliations:** 1 Faculty of Teacher Education, Weifang University, Weifang, China; 2 School of Psychology, Shandong Second Medical University, Weifang, China; 3 Neonatal Department, Weifang People's Hospital, Weifang, China

**Keywords:** attention, feature-sharing distractors, perceptual interference, pre-cues, retro-cues, visual working memory

## Abstract

Visual working memory (VWM) is a capacity-limited system that enables visual information to be maintained, prioritized, and used after perceptual input has disappeared. Although attention is often assumed to protect VWM from distraction, recent work suggests that distraction is not uniformly harmful and that its impact depends on the representational and attentional state of the memorized information. The present study examined how attentional prioritization and distractor similarity jointly shape VWM across encoding and maintenance. In two change-detection experiments, cue load and distractor type were manipulated within participants. Experiment 1 used pre-cues to guide external attention before encoding, whereas Experiment 2 used retro-cues to guide internal attention during maintenance. Trial-level binomial generalized linear mixed-effects models for accuracy and linear mixed-effects models for log-transformed correct-trial reaction time showed that performance was best when one or two items were prioritized and declined when attention was distributed across more items or when no cue was provided. Distractor effects depended on cue condition. Under broad or absent attentional guidance, non-feature-sharing distractors were generally more disruptive than distractors that shared one feature with an unprobed memory item; however, feature-sharing distractors did not improve accuracy relative to the no-distractor baseline. Accuracy and reaction time converged for the cue-load effect but often dissociated for distractor contrasts, indicating that RT-only effects should be interpreted cautiously as changes in retrieval or decision efficiency rather than memory fidelity. These findings support a conditional account in which the effect of feature overlap depends on attentional allocation and the accessibility of the maintained representations.

## 1 Introduction

Visual working memory (VWM) enables visual information to remain available for comparison, decision making, and goal-directed action after the sensory input has disappeared. Classic change-detection work established that VWM is highly capacity limited, but the nature of this limit remains debated (Luck and Vogel, 1997; Vogel et al., 2001). Slot-based and item-limit accounts emphasize that only a small number of object representations can be maintained with high fidelity (Adam et al., 2017; Cowan, 2001; Luck and Vogel, 2013), whereas resource-based accounts propose that memory quality depends on how a limited representational resource is distributed across items (Bays and Husain, 2008). More recent perspectives emphasize that VWM capacity cannot be understood only in terms of item number, because memory performance is also shaped by feature binding, ensemble structure, task demands, and the accessibility of stored representations (Baddeley, 2000, 2012; Brady et al., 2011; Hitch et al., 2020).

Attention is central to this system because it determines which information is encoded, maintained, and retrieved. External attention selects information from the sensory environment, whereas internal attention prioritizes information already held in mind (Chun et al., 2011; Gazzaley and Nobre, 2012; Kiyonaga and Egner, 2013). The close relationship between attention and working memory is supported by evidence for overlapping spatial mechanisms, shared resource demands, and common frontoparietal control processes (Awh and Jonides, 2001; Awh et al., 2006; van Ede and Nobre, 2023). At the same time, external and internal attention are not identical. They operate over different representational domains and may differ in timing, selection costs, and vulnerability to interference (Nobre and Gresch, 2025; Nobre and van Ede, 2023; van Ede, 2020).

Pre-cue and retro-cue paradigms provide a powerful way to separate these attentional operations. Pre-cues are presented before the memory array and can improve perceptual selection and encoding efficiency, whereas retro-cues are presented after encoding and can prioritize stored information, improve retrieval, and protect memory from subsequent interference (Griffin and Nobre, 2003; Lepsien et al., 2005; Makovski et al., 2008; Souza et al., 2016). A large body of research has shown that retro-cues can improve VWM even when the stimulus is no longer present, suggesting that attention can be oriented within internal representations in a manner partly analogous to spatial orienting in perception (Astle et al., 2012; Rerko et al., 2014; Souza and Oberauer, 2016). However, retro-cue benefits are not automatic or cost free. They can depend on central attentional capacity, cue-probe timing, and whether attention is directed to items or feature dimensions (Hajonides et al., 2020; Janczyk and Berryhill, 2014; Ye et al., 2016).

These limitations raise an important question: how many representations can attention effectively prioritize at one time? Some theories assume that several chunks can be maintained within the focus of attention, whereas others propose a narrow focus that selects a single item at a time (Cowan, 2011; Oberauer, 2002). Contemporary accounts often reconcile these positions by distinguishing among degrees of accessibility rather than assuming a single binary distinction between attended and unattended memory states (Myers et al., 2017; Oberauer, 2019; Oberauer and Hein, 2012). From this perspective, increasing the number of cued items may reduce the quality of prioritization for each item, thereby weakening attentional protection and increasing susceptibility to distraction (Allen and Ueno, 2018; Lin et al., 2021).

Distraction provides a sensitive test of how robust VWM representations are. Earlier work often treated distraction as a purely harmful input that competes with maintained information, but recent evidence indicates that VWM can resist distraction through multiple mechanisms, including selective gating, prioritization, temporal expectation, and active shielding (Lorenc et al., 2021). At the same time, prioritization does not confer uniform protection across tasks; prioritized representations can be protected, unaffected, or vulnerable depending on how priority and interference are implemented (Allen and Ueno, 2018; Vergauwe et al., 2025; Zhang and Lewis-Peacock, 2023a,b). Formal interference models of VWM further treat change-detection decisions as competition among noisy item representations and context-bound candidates, so the effect of a distractor depends on both representational similarity and the accessibility of the competing traces (Lin and Oberauer, 2022; Oberauer and Lin, 2017).

Recent signal-intrusion accounts likewise propose that a distractor-derived signal can enter the memory-based decision process, producing different behavioral consequences as a function of target-distractor similarity and memory strength (Zhang and Lewis-Peacock, 2025). This framework does not imply that feature-sharing distraction should improve memory. In the present task, feature-sharing distractors matched the color or shape of an unprobed memory item. Therefore, an advantage over non-feature-sharing distractors could reflect reduced novelty or mismatch, greater consistency with the feature context of the memory array, or less disruptive signal intrusion, rather than strengthening of the probed representation. Conversely, overlapping input could increase ambiguity or binding competition when several representations remain accessible (Czoschke et al., 2023; Shepherdson et al., 2021; Wennberg and Serences, 2024). The no-distractor condition is therefore essential for distinguishing true facilitation from reduced interference relative to an unrelated distractor.

Despite these developments, it remains unclear how the effect of distractor similarity changes as the number of prioritized items increases, and whether this relationship differs between external and internal attention. The present study addressed this question by jointly manipulating cue load and distractor similarity across two experiments. Experiment 1 used pre-cues to manipulate external attention before encoding. Experiment 2 used retro-cues to manipulate internal attention during maintenance. In both experiments, distractors either shared a feature with one of the memory items, shared no features with the memory array, or were absent. The primary outcome was recognition accuracy, which indexes whether the memory representation supported a correct old/new decision. Correct-trial reaction time was analyzed as a complementary measure of retrieval and decision efficiency. We made two confirmatory predictions. First, cueing should improve accuracy and reaction time most strongly when one or two items are prioritized, with weaker benefits as cue load increases. Second, non-feature-sharing distractors should generally be more disruptive than feature-sharing distractors when attentional guidance is weak or diffuse.

## 2 Experiment 1

Experiment 1 examined how external attention protects VWM representations from distraction during encoding. Pre-cues were used to direct attention to one or more likely target locations before the memory array appeared, thereby manipulating the breadth of attentional prioritization. During the retention interval, a visual distractor was presented to test whether the robustness of the encoded representations depended on cue load and distractor similarity. We expected focused pre-cues to produce stronger protection than diffuse or absent cueing, and we expected feature-sharing distractors to be less disruptive than non-feature-sharing distractors when attentional guidance was weak.

### 2.1 Methods

#### 2.1.1 Participants

An *a priori* power analysis was conducted using G^*^Power 3.1 (Faul et al., 2009) to determine the required sample size. Assuming a medium effect size (*f* = 0.25), an alpha level of 0.05, and statistical power of 0.95 for a 5 × 3 within-subjects design, the analysis indicated that a minimum of 16 participants was required. To ensure adequate statistical power and maintain comparability with previous studies using similar visual working memory paradigms (Allen and Ueno, 2018), 21 undergraduate students were recruited from Shandong Second Medical University. The final sample consisted of 18 participants (*M*_age_ = 21.94 years, SD = 1.11; 13 female participants). All participants were native Chinese speakers, reported normal or corrected-to-normal vision, and had no color blindness. Written informed consent was obtained from all participants before the experiment. The study was approved by the Ethics Committee of Shandong Second Medical University and was conducted in accordance with the approved protocol. Participants received 10 RMB as compensation.

#### 2.1.2 Equipment and materials

The experiment was programmed and administered using E-Prime 2.0. Stimuli were presented on a 15.6-in. Dell monitor with a resolution of 1,024 × 768 pixels and a refresh rate of 60 Hz. Participants were seated approximately 60 cm from the screen. Each memory display contained four colored shapes presented against a white background (RGB = 255, 255, 255). Each shape subtended approximately 1.5 ° × 1.5 ° of visual angle. On each trial, four items were randomly selected without replacement from a pool of 64 unique color-shape combinations. The stimulus pool was created by combining eight colors (blue [RGB = 0, 0, 255], gray [RGB = 128, 128, 128], green [RGB = 0, 255, 0], black [RGB = 0, 0, 0], yellow [RGB = 255, 255, 0], cyan [RGB = 65, 224, 208], red [RGB = 255, 0, 0], and purple [RGB = 128, 0, 128]) with eight shapes (diamond, cross, flag, V-shape, pentagon, circle, triangle, and arch).

#### 2.1.3 Experimental design

Experiment 1 employed a within-subjects design in which Cue Type and Distractor Type were manipulated as repeated-measures factors. Cue Type comprised five levels: one-cue, two-cue, three-cue, four-cue, and no-cue. Distractor Type comprised three levels: feature-sharing distractor, non-feature-sharing distractor, and no-distractor. The dependent variables were accuracy and reaction time on the recognition task. Pre-cues consisted of black arrows presented immediately prior to the memory array and were used to manipulate the distribution of attentional resources during encoding. In the no-cue condition, no spatial information was provided, and each of the four memory items was equally likely to be tested. In the one-, two-, three-, and four-cue conditions, the probe was always selected from the set of cued items. Accordingly, the conditional probability that any individual cued item would be probed was 1.00, 0.50, 0.33, and 0.25, respectively. Items that were not cued were never tested. Distractor stimuli were categorized according to their degree of featural overlap with the memorized items. Feature-sharing distractors matched either the color or the shape of one of the unprobed memory items, whereas non-feature-sharing distractors shared neither color nor shape with any item in the memory array. In the no-distractor condition, no stimulus was presented during the interference interval, and the central fixation cross remained on the screen.

#### 2.1.4 Experimental procedure

**Figure 1:**
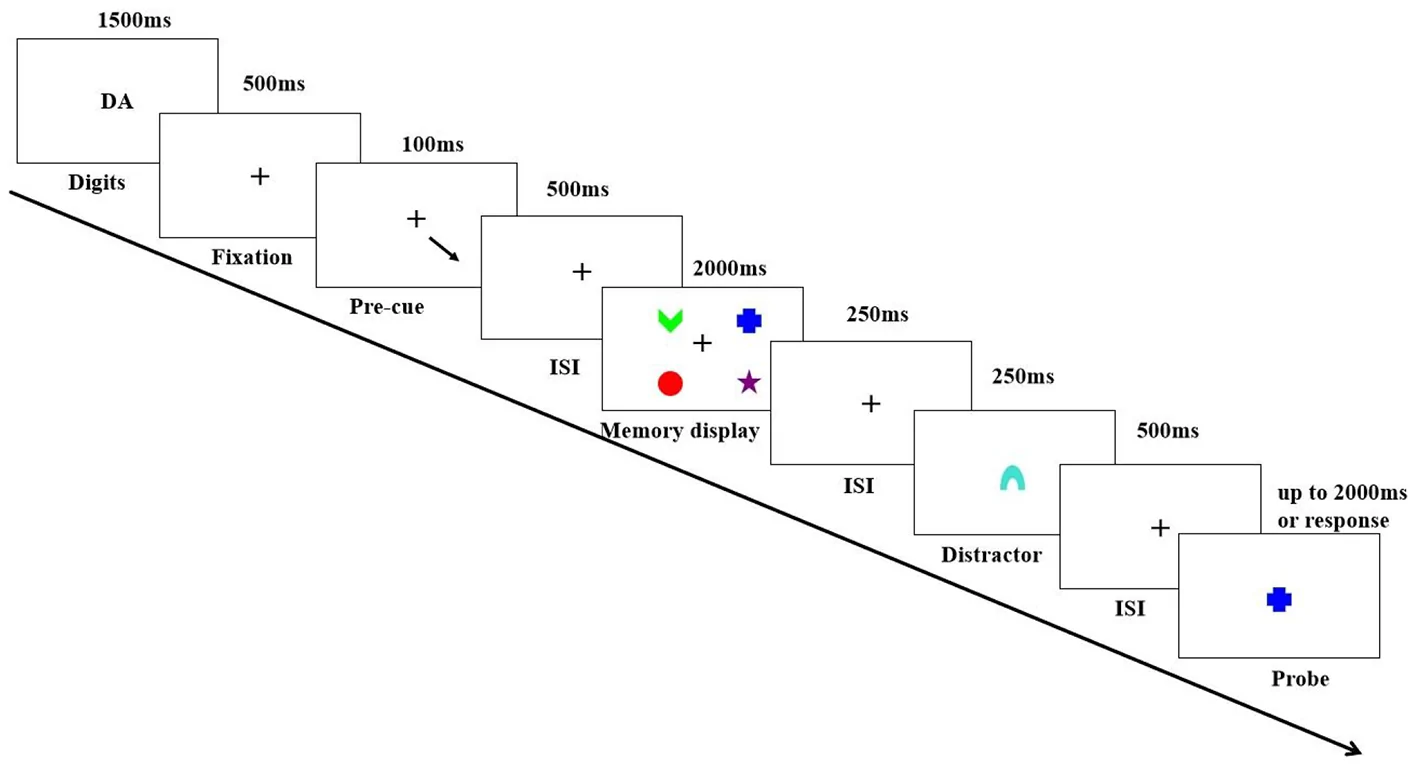
Trial procedure for Experiment 1. Each trial began with two digits (1,500 ms), followed by fixation (500 ms), a pre-cue or no-cue display (100 ms), a 500-ms interstimulus interval, and the memory array (2,000 ms). After memory-array offset, fixation was shown for 250 ms, followed by a distractor or fixation-only control (250 ms) and a final retention interval (500 ms). A single centered probe was then displayed for up to 2,000 ms or until response.

The trial sequence is illustrated in Figure 1. Each trial began with the presentation of two digits for 1,500 ms. Participants were instructed to rehearse these digits throughout the trial to impose articulatory suppression and thereby reduce verbal encoding of the visual stimuli. A central fixation cross was then presented and remained visible throughout the trial except during stimulus presentation. Following a 500-ms fixation interval, the pre-cue display was presented for 100 ms. In the cue conditions, one to four black arrows spatially indicated the locations of the memory items that were most likely to be tested. In the no-cue condition, only the fixation cross was displayed. After a 500-ms interstimulus interval, the memory array was presented for 2,000 ms. The memory array consisted of four colored shapes positioned around fixation. After the memory array disappeared, fixation remained for 250 ms, after which a distractor stimulus was presented for 250 ms in the distractor conditions. Feature-sharing distractors matched either the color or the shape of one of the non-probed memory items, whereas non-feature-sharing distractors shared no features with any item in the memory array. In the no-distractor condition, fixation remained throughout this 250-ms interval. The distractor, or the corresponding fixation interval, was followed by a final 500-ms retention interval. Subsequently, a single probe item was presented at the center of the screen and remained visible for up to 2,000 ms or until a response was made. Participants were instructed to indicate whether the probe item had been present in the preceding memory array by pressing one of two response keys. The assignment of “yes” and “no” responses was counterbalanced across participants. The experiment consisted of 360 experimental trials divided into six blocks of 60 trials each. Cue Type and Distractor Type were pseudo-randomized within each block. Participants were allowed short rest breaks between blocks. The entire experimental session lasted approximately 40 min.

#### 2.1.5 Data analysis

Accuracy and reaction time were analyzed at the trial level in R version 4.4.2 (R Core Team, 2024). Accuracy was analyzed using binomial generalized linear mixed-effects models (GLMMs) with a logit link. Reaction time was analyzed on correct trials with positive reaction times using linear mixed-effects models (LMMs) fitted to log-transformed reaction time (Barr et al., 2013). Cue Type (one-cue, two-cue, three-cue, four-cue, and no-cue), Distractor Type (feature-sharing, non-feature-sharing, and no-distractor), and their interaction were specified as fixed effects. Participant was included as a random intercept to account for repeated observations within individuals. Models were fitted using lme4 (Bates et al., 2015), and follow-up comparisons were conducted using estimated marginal means with Holm correction (Lenth, 2023). Nested models were compared using likelihood-ratio tests under maximum-likelihood estimation. Accuracy contrasts are reported on the log-odds scale with odds ratios where informative, and reaction-time contrasts are reported on the log-ratio scale. Descriptive means and standard errors of the mean (SEMs) were calculated across participants, whereas model-based figures display estimated marginal means with 95% confidence intervals.

### 2.2 Results

#### 2.2.1 Accuracy

**Figure 2:**
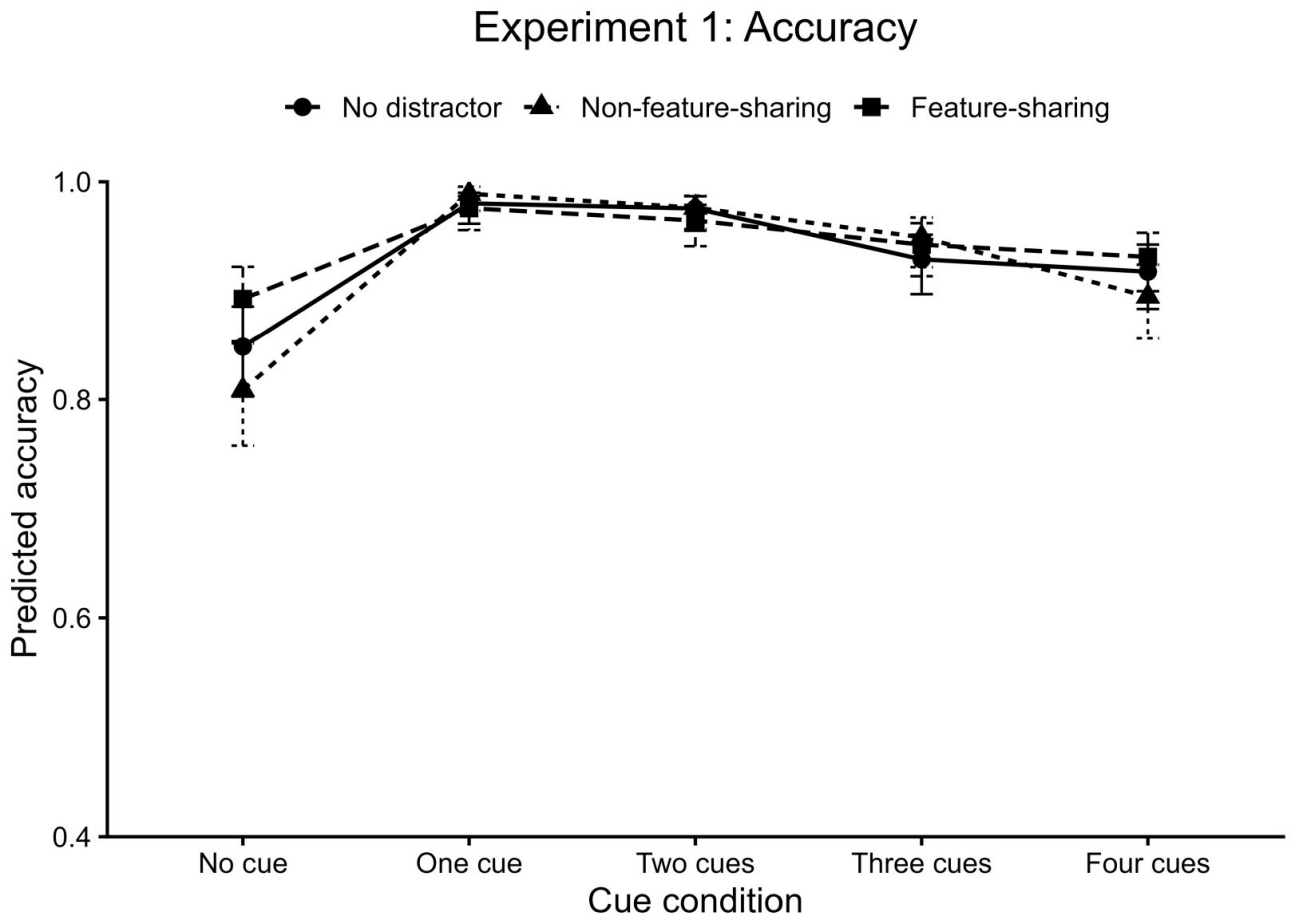
Model-estimated recognition accuracy in Experiment 1 as a function of Cue Type and Distractor Type. Error bars represent 95% confidence intervals from the fitted binomial GLMM.

Accuracy across conditions in Experiment 1 is presented in Figure 2. The binomial generalized linear mixed-effects model included 6,360 trial-level observations from 18 participants. Likelihood-ratio tests showed a significant effect of Cue Type, $\chi_{\left(4\right)}^{2}$ = 228.33, *p* < 0.001, whereas the additive effect of Distractor Type was not significant, $\chi_{\left(2\right)}^{2}$ = 5.46, *p* = 0.065. Follow-up comparisons of the Cue Type effect showed that accuracy was highest when one or two items were prioritized. Accuracy in the one- and two-cue conditions exceeded that in the three-, four-, and no-cue conditions (*ps* < 0.001), whereas the difference between the one- and two-cue conditions was not significant (*p* > 0.05). Accuracy further declined from the three-cue to the four-cue and no-cue conditions.

Importantly, adding the Cue Type × Distractor Type interaction significantly improved model fit, $\chi_{\left(8\right)}^{2}$ = 15.79, *p* = 0.046, indicating that the effect of distraction varied across cue conditions. Simple-effects analyses of the interaction showed that Distractor Type significantly affected accuracy only in the no-cue condition, $\chi_{\left(2\right)}^{2}$ = 11.93, *p* = 0.003. When no pre-cue was provided, accuracy was significantly higher in the feature-sharing distractor condition than in the non-feature-sharing distractor condition, *b* = 0.67, SE = 0.20, OR = 1.95, *z* = 3.40, Holm-adjusted *p* = 0.002. The no-distractor condition did not differ significantly from the non-feature-sharing distractor condition, *b* = 0.28, SE = 0.18, *z* = 1.55, OR = 1.32, Holm-adjusted *p* = 0.122, or from the feature-sharing condition, *b* = −0.39, SE = 0.20, *z* = −1.92, OR = 0.68, Holm-adjusted *p* = 0.109. Distractor Type did not significantly affect accuracy in the one-cue condition, $\chi_{\left(2\right)}^{2}$ = 2.22, *p* = 0.329; the two-cue condition, $\chi_{\left(2\right)}^{2}$ = 1.45, *p* = 0.485; the three-cue condition, $\chi_{\left(2\right)}^{2}$ = 1.68, *p* = 0.432; or the four-cue condition, $\chi_{\left(2\right)}^{2}$ = 3.79, *p* = 0.151. These results suggest that focusing attention on a limited number of items generally enhances accuracy. However, in the absence of attentional prioritization cues, feature-sharing distractors yielded higher accuracy than non-feature-sharing distractors.

#### 2.2.2 Reaction time

**Figure 3:**
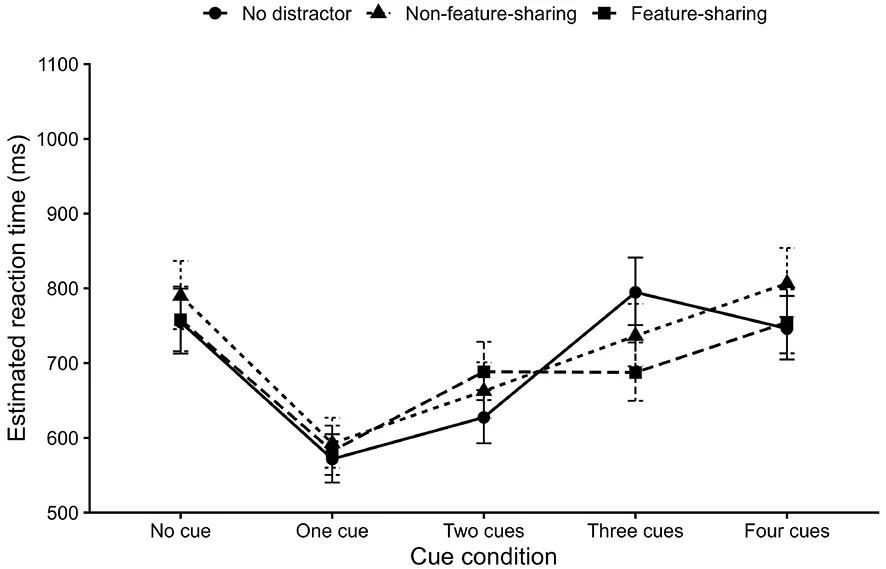
Model-estimated reaction time in Experiment 1 as a function of Cue Type and Distractor Type. Estimates were back-transformed from the log-reaction time LMM and are shown in milliseconds. Error bars represent 95% confidence intervals from the fitted model.

Mean reaction times across conditions in Experiment 1 are presented in Figure 3. The analysis included 5,902 correct trials with valid positive response times. Reaction times were log transformed, and estimated marginal means were back transformed into milliseconds for presentation. Likelihood ratio tests revealed a significant effect of Cue Type, $\chi_{\left(4\right)}^{2}$ = 950.05, *p* < 0.001. Reaction times were fastest in the one-cue condition, followed by the two-cue and three-cue conditions (*ps* < 0.001), whereas the no-cue and four-cue conditions were slowest and did not differ significantly from each other (*p* > 0.05).

Adding Distractor Type significantly improved model fit, $\chi_{\left(2\right)}^{2}$ = 16.06, *p* < 0.001. More importantly, the Cue Type × Distractor Type interaction also improved model fit, $\chi_{\left(8\right)}^{2}$ = 104.64, *p* < 0.001. Simple-effects analyses showed significant effects of Distractor Type in the no-cue [$\chi_{\left(2\right)}^{2}$ = 6.65, *p* = 0.036], two-cue [$\chi_{\left(2\right)}^{2}$ = 27.07, *p* < 0.001], three-cue [$\chi_{\left(2\right)}^{2}$ = 63.42, *p* < 0.001], and four-cue [$\chi_{\left(2\right)}^{2}$ = 20.67, *p* < 0.001] conditions, but not in the one-cue condition [$\chi_{\left(2\right)}^{2}$ = 4.14, *p* = 0.126]. In the no-cue condition, none of the pairwise comparisons remained significant after Holm correction: non-feature-sharing vs. no distractor, *b* = 0.05, SE = 0.02, *z* = 2.33, ratio = 1.05, Holm-adjusted *p* = 0.059; feature-sharing vs. no distractor, *b* = 0.004, SE = 0.02, *z* = 0.21, ratio = 1.00, Holm-adjusted *p* = 0.834; and non-feature-sharing vs. feature-sharing, *b* = 0.04, SE = 0.02, *z* = 2.15, ratio = 1.04, Holm-adjusted *p* = 0.063.

In the two-cue condition, both non-feature-sharing and feature-sharing distractors slowed responses relative to the no-distractor condition, *b* = 0.05, SE = 0.02, *z* = 3.07, ratio = 1.06, Holm-adjusted *p* = 0.004, and *b* = 0.09, SE = 0.02, *z* = 5.17, ratio = 1.10, Holm-adjusted *p* < 0.001, respectively. Feature-sharing distractors also produced slower responses than non-feature-sharing distractors, *b* = 0.04, SE = 0.02, *z* = 2.15, ratio = 1.04, Holm-adjusted *p* = 0.031.

In the three-cue condition, both non-feature-sharing and feature-sharing distractors produced faster responses than the no-distractor condition, *b* = −0.08, SE = 0.02, *z* = −4.19, ratio = 0.93, Holm-adjusted *p* < 0.001, and *b* = −0.14, SE = 0.02, *z* = −7.96, ratio = 0.87, Holm-adjusted *p* < 0.001, respectively. Responses were also faster following feature-sharing than non-feature-sharing distractors, *b* = −0.07, SE = 0.02, *z* = −3.80, ratio = 0.93, Holm-adjusted *p* < 0.001.

In the four-cue condition, non-feature-sharing distractors slowed responses relative to both the no-distractor condition, *b* = 0.08, SE = 0.02, *z* = 4.21, ratio = 1.08, Holm-adjusted *p* < 0.001, and the feature-sharing condition, *b* = 0.07, SE = 0.02, *z* = 3.61, ratio = 1.07, Holm-adjusted *p* < 0.001. The feature-sharing and no-distractor conditions did not differ significantly, *b* = 0.01, SE = 0.02, *z* = 0.62, ratio = 1.01, Holm-adjusted *p* = 0.533. These results indicate that reaction times increased with cue load, while distractor effects varied across conditions: distractors generally slowed responses, although feature overlap reduced interference relative to non-overlapping or no-distractor conditions. However, when three items were cued, distractors may have acted as additional attentional cues, thereby facilitating response speed.

### 2.3 Discussion

Experiment 1 demonstrated a clear capacity limit on attentional prioritization in VWM. Accuracy was highest when attention was directed to one or two items and declined significantly as the number of cued items increased. Reaction times showed the same general pattern, with faster responses under narrowly focused attention and slower responses as cue load broadened. These converging effects indicate that the benefit of external prioritization was graded rather than all-or-none: attention was most effective when concentrated on a small subset of representations, whereas distributing priority across more items reduced the benefit afforded to each item. This pattern is consistent with resource-based and priority-state accounts, which propose that limited attentional and representational resources are shared among prioritized items (Bays and Husain, 2008; Cowan, 2011; Oberauer, 2019). Thus, prioritizing one or two items likely strengthened their accessibility and precision, whereas broader prioritization diluted attentional weighting and weakened performance.

The interaction between cue load and distraction further showed that resistance to interference depended on the current attentional state. When attention was narrowly focused, especially in the one-cue condition, distractors produced no reliable cost, indicating that strongly prioritized representations were relatively resistant to irrelevant sensory input. In the no-cue condition, accuracy was higher for feature-sharing than for non-feature-sharing distractors. However, neither distractor condition differed from the no-distractor baseline, and no reliable reaction-time differences emerged. This pattern therefore does not indicate memory facilitation. Rather, it suggests that feature-sharing distractors were less disruptive than unrelated distractors when no representation had been selectively prioritized. One possibility is that distractors sharing a feature with the memory array generated less novel or less discrepant evidence than non-overlapping distractors, thereby producing weaker interference under diffuse attentional states (Lorenc et al., 2021; Zhang and Lewis-Peacock, 2025). The present design, however, cannot determine whether this relative sparing arose during maintenance, retrieval, or recognition decisions.

The reaction-time effects at intermediate cue loads revealed a different pattern. In the two-cue condition, both distractor types slowed correct responses relative to the no-distractor condition, with the greatest slowing produced by feature-sharing distractors, while accuracy remained unchanged. This dissociation suggests that distraction increased retrieval or decision demands without degrading the stored representations themselves. Feature overlap may have produced additional ambiguity because the distractor partially matched an accessible memory item, thereby delaying selection of the correct response. By contrast, in the three-cue condition, both distractor types accelerated responses without improving accuracy. This isolated reaction-time benefit should not be interpreted as enhanced memory. A more plausible explanation is temporal alerting: because the distractor was a salient event presented at a fixed interval before the probe, it may have acted as a warning signal that facilitated response preparation while leaving memory fidelity unchanged (van Ede et al., 2017, 2018).

Overall, Experiment 1 shows that attentional prioritization enhances VWM most effectively when restricted to a small number of items. As prioritization broadens, performance declines and distractor effects become more variable, indicating that attentional protection is limited, graded, and sensitive to both cue load and feature similarity.

## 3 Experiment 2

Experiment 2 examined whether internally directed attention provides comparable protection after encoding has occurred. Retro-cues were presented during the retention interval to prioritize selected memory representations. This design allowed us to test whether the effect of distractor similarity depends on the state of internal attention during maintenance. We expected focused retro-cues to reduce distractor costs when cue load was low. Effects observed only in reaction time, including patterns compatible with competition or facilitation, were treated as exploratory because reaction time can reflect retrieval, decision, temporal preparation, and motor processes in addition to memory quality.

### 3.1 Method

#### 3.1.1 Participants

A total of 22 undergraduate students were recruited from Shandong Second Medical University. Two participants withdrew before completing the experiment, leaving a final sample of 20 participants (*M*_age_ = 21.80 years, SD = 1.24; 14 female participants). All participants were native Chinese speakers, had normal or corrected-to-normal vision, and reported no color blindness. Written informed consent was obtained before participation. The study was approved by the Ethics Committee of Shandong Second Medical University. Participants received 10 RMB as compensation.

#### 3.1.2 Equipment and materials

The equipment and materials were identical to those used in Experiment 1.

#### 3.1.3 Experimental design and procedure

Experiment 2 used a 5 × 3 within-subjects design, with Cue Type and Distractor Type as repeated-measures factors. Cue Type included five levels: one-cue, two-cue, three-cue, four-cue, and no-cue. Distractor Type included three levels: feature-sharing distractor, non-feature-sharing distractor, and no-distractor. The cue manipulation was identical to that in Experiment 1, except that retro-cues were presented after the memory array and during the retention interval rather than before encoding. The distractor manipulation was also identical to that in Experiment 1.

**Figure 4:**
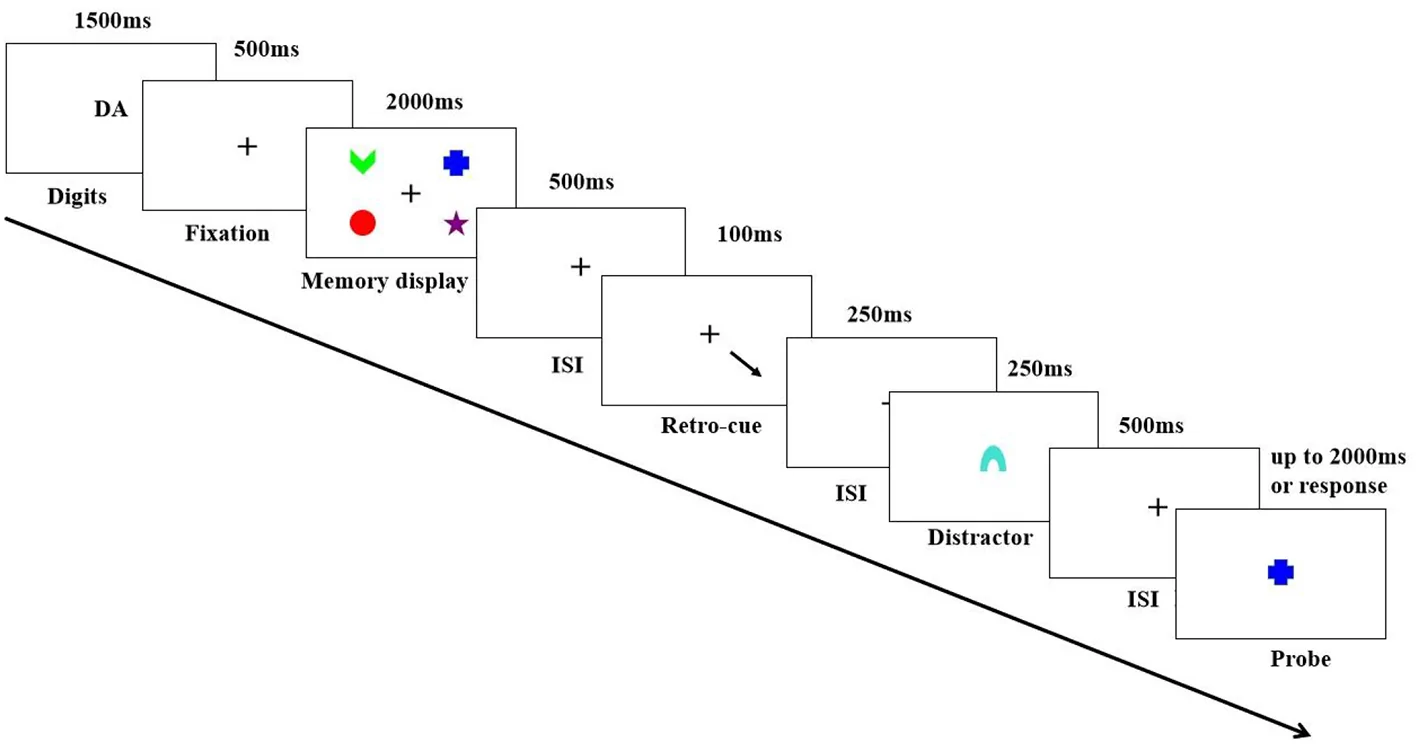
Trial procedure for Experiment 2. Each trial began with two digits (1,500 ms), fixation (500 ms), and the memory array (2,000 ms). After a 500-ms delay, a retro-cue or no-cue display was presented (100 ms), followed by another 500-ms delay, a distractor or fixation-only control (250 ms), and a final retention interval (500 ms). A single centered probe was then displayed for up to 2,000 ms or until response.

The trial sequence is shown in Figure 4. Each trial began with the presentation of two digits for 1,500 ms. Participants were instructed to rehearse these digits throughout the trial to reduce verbal recoding of the visual stimuli. A black fixation cross was then presented at the center of the screen. After a 500-ms fixation interval, the memory array appeared for 2,000 ms. Following a 500-ms delay, the retro-cue display was presented for 100 ms. In the cue conditions, one to four black arrows indicated the locations of the memory items that were most likely to be tested. In the no-cue condition, only the fixation cross was presented. After another 500-ms delay, a distractor was presented for 250 ms in the distractor conditions; in the no-distractor condition, only the fixation cross remained on the screen. After a final 500-ms retention interval, a single probe item was presented at the center of the screen for up to 2,000 ms or until a response was made. Participants were asked to answer whether the probe item had appeared in the preceding memory array by pressing one of two response keys. “Yes” and “no” trials occurred with equal probability, and response-key assignment was counterbalanced across participants. The experiment consisted of 360 trials divided into six blocks of 60 trials. Cue Type and Distractor Type were pseudorandomized within each block. Participants were informed that the retro-cues were valid and were allowed short rest breaks between blocks. The entire experimental session lasted approximately 40 min.

#### 3.1.4 Analysis

Accuracy and reaction time were analyzed at the trial level using the same binomial GLMM and log-reaction-time LMM specifications as in Experiment 1. Cue Type, Distractor Type, and their interaction were entered as fixed effects, and Participant was included as a random intercept. Significant interactions were followed by simple-effects analyses of Distractor Type within each cue condition, with Holm-adjusted pairwise contrasts used for theoretically informative comparisons.

### 3.2 Results

#### 3.2.1 Accuracy

**Figure 5:**
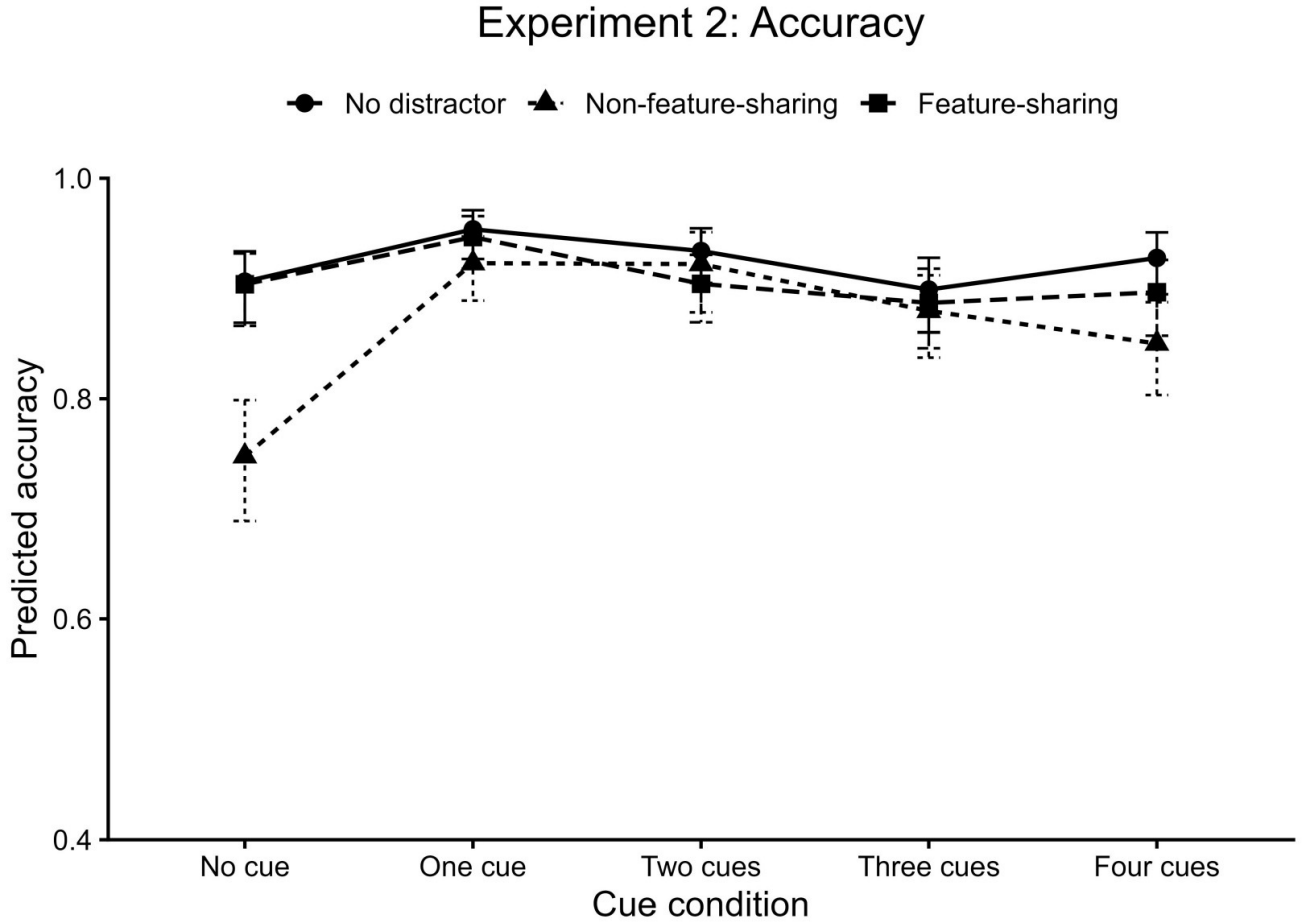
Model-estimated recognition accuracy in Experiment 2 as a function of Cue Type and Distractor Type. Error bars represent 95% confidence intervals from the fitted binomial GLMM.

Accuracy across conditions in Experiment 2 is presented in Figure 5. The binomial generalized linear mixed-effects model included 6,000 trial-level observations from 20 participants. Likelihood-ratio tests showed a significant effect of Cue Type, $\chi_{\left(4\right)}^{2}$ = 61.90, *p* < 0.001. Accuracy was highest when one or two items were retro-cued and generally declined as internal attention was distributed across more items or was not guided by a retro-cue. Adding Distractor Type significantly improved model fit, $\chi_{\left(2\right)}^{2}$ = 43.79, *p* < 0.001. Importantly, the Cue Type × Distractor Type interaction also significantly improved model fit, $\chi_{\left(8\right)}^{2}$ = 26.76, *p* < 0.001, indicating that the effect of distraction varied across retro-cue conditions.

Simple-effects analyses of the interaction showed significant distractor effects in the four-cue condition, $\chi_{\left(2\right)}^{2}$ = 12.68, *p* = 0.002, and the no-cue condition, $\chi_{\left(2\right)}^{2}$ = 49.84, *p* < 0.001. Distractor Type did not significantly affect accuracy in the one-cue condition, $\chi_{\left(2\right)}^{2}$ = 3.92, *p* = 0.141, the two-cue condition, $\chi_{\left(2\right)}^{2}$ = 3.05, *p* = 0.218, or the three-cue condition, $\chi_{\left(2\right)}^{2}$ = 0.80, *p* = 0.670. Specifically, in the no-cue condition, accuracy was significantly higher in the no-distractor condition than in the non-feature-sharing condition, *b* = 1.18, SE = 0.20, *z* = 5.80, OR = 3.25, Holm-adjusted *p* < 0.001. Accuracy was also significantly higher following feature-sharing distractors than following non-feature-sharing distractors, *b* = 1.15, SE = 0.20, *z* = 5.70, OR = 3.16, Holm-adjusted *p* < 0.001. However, the no-distractor and feature-sharing conditions did not differ significantly, *b* = 0.03, SE = 0.24, *z* = 0.12, OR = 1.03, Holm-adjusted *p* = 0.906.

In the four-cue condition, accuracy was significantly higher in the no-distractor condition than in the non-feature-sharing condition, *b* = 0.81, SE = 0.23, *z* = 3.49, OR = 2.26, Holm-adjusted *p* = 0.001. The no-distractor and feature-sharing conditions did not differ significantly, *b* = 0.39, SE = 0.25, *z* = 1.59, OR = 1.48, Holm-adjusted *p* = 0.112. No significant differences were found between the accuracy following feature-sharing distractors and following non-feature-sharing distractors, *b* = 0.42, SE = 0.21, *z* = 1.99, OR = 1.53, Holm-adjusted *p* = 0.093.

Overall, recognition accuracy was highest when only a small number of items was prioritized and declined as attention was distributed more broadly. Distractor effects depended on cue condition: significant accuracy costs were observed only in the no-cue and four-cue conditions, where non-feature-sharing distractors reduced accuracy relative to the no-distractor condition. In contrast, distractor type did not reliably affect accuracy when one, two, or three items were retro-cued.

#### 3.2.2 Reaction time

**Figure 6:**
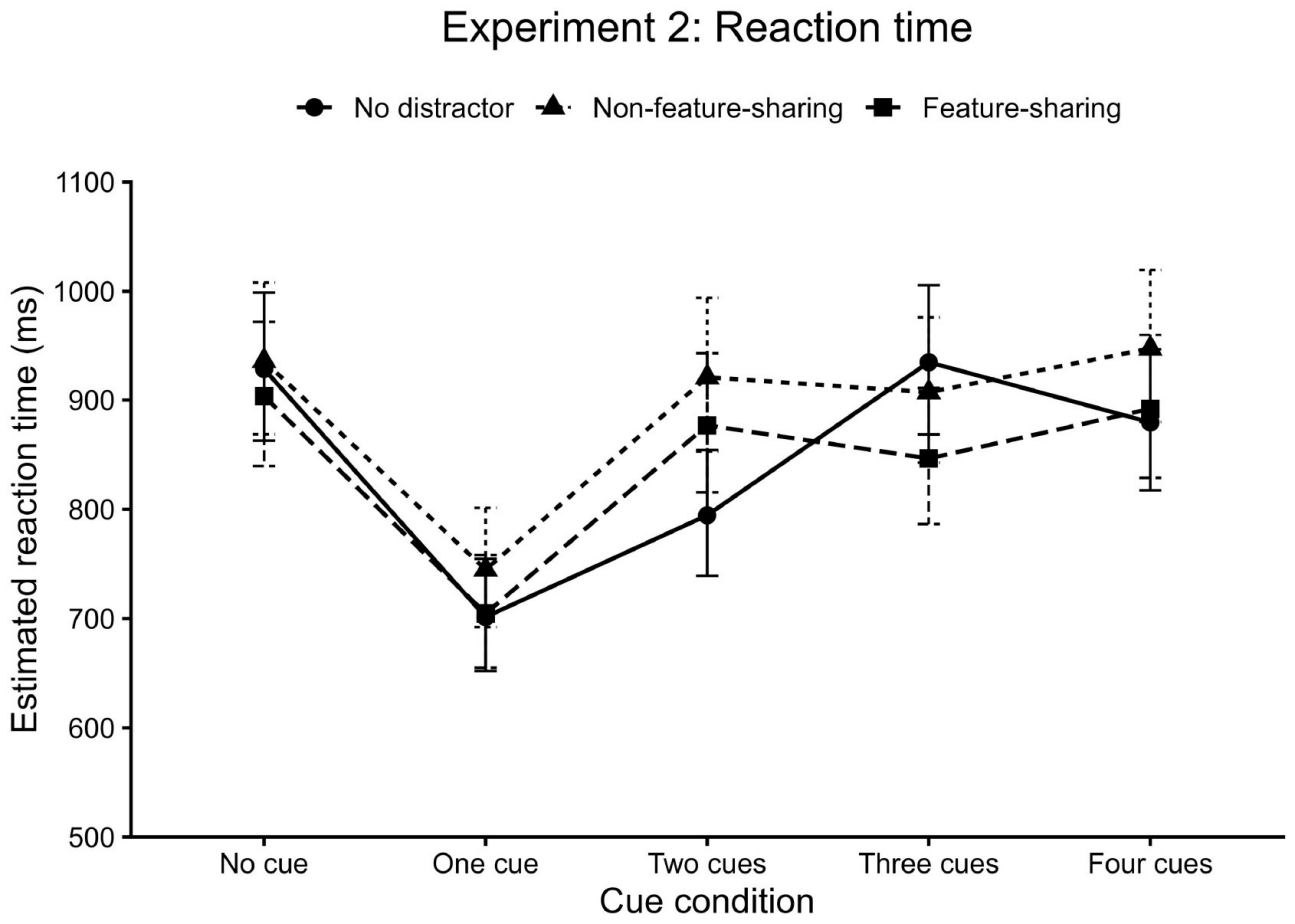
Model-estimated reaction time in Experiment 2 as a function of Cue Type and Distractor Type. Estimates were back-transformed from the log-reaction time LMM and are shown in milliseconds. Error bars represent 95% confidence intervals from the fitted model.

Mean reaction times across conditions are presented in Figure 6. The reaction-time analysis included 5,356 correct trials with valid positive response times. Reaction times were log-transformed prior to analysis, and the estimated marginal means were subsequently back-transformed into milliseconds for presentation. Likelihood-ratio tests revealed a significant main effect of Cue Type, $\chi_{\left(4\right)}^{2}$ = 611.78, *p* < 0.001. Responses were fastest in the one-cue condition, followed by the two-cue and three-cue conditions (*ps* < 0.001), whereas responses were slowest in the no-cue and four-cue conditions. Adding Distractor Type significantly improved model fit, $\chi_{\left(2\right)}^{2}$ = 40.40, *p* < 0.001. More importantly, the Cue Type × Distractor Type interaction also significantly improved model fit, $\chi_{\left(8\right)}^{2}$ = 69.72, *p* < 0.001, indicating that the effect of distractor type varied across cue conditions.

Simple-effects analyses showed significant distractor effects in the one-cue [$\chi_{\left(2\right)}^{2}$ = 11.75, *p* = 0.003], two-cue [$\chi_{\left(2\right)}^{2}$ = 54.91, *p* < 0.001], three-cue [$\chi_{\left(2\right)}^{2}$ = 25.99, *p* < 0.001], and four-cue [$\chi_{\left(2\right)}^{2}$ = 15.30, *p* < 0.001] conditions, but not in the no-cue condition, $\chi_{\left(2\right)}^{2}$ = 3.28, *p* = 0.194. In the one-cue condition, non-feature-sharing distractors produced slower responses than the no-distractor condition, *b* = 0.060, SE = 0.019, *z* = 3.09, ratio = 1.06, Holm-adjusted *p* = 0.006. Responses were also slower following non-feature-sharing distractors than following feature-sharing distractors, *b* = 0.055, SE = 0.019, *z* = 2.85, ratio = 1.06, Holm-adjusted *p* = 0.009. The feature-sharing and no-distractor conditions did not differ, *b* = 0.005, SE = 0.019, *z* = 0.23, ratio = 1.00, Holm-adjusted *p* = 0.815.

In the two-cue condition, both distractor types slowed responses relative to the no-distractor baseline. Non-feature-sharing distractors produced slower responses than the no-distractor condition, *b* = 0.147, SE = 0.022, *z* = 6.76, ratio = 1.16, Holm-adjusted *p* < 0.001. Feature-sharing distractors also slowed responses relative to the no-distractor condition, *b* = 0.099, SE = 0.018, *z* = 5.51, ratio = 1.10, Holm-adjusted *p* < 0.001. In addition, responses were slower following non-feature-sharing distractors than following feature-sharing distractors, *b* = 0.049, SE = 0.022, *z* = 2.23, ratio = 1.05, Holm-adjusted *p* = 0.026.

In the three-cue condition, responses were significantly faster following feature-sharing distractors than in the no-distractor condition, *b* = −0.099, SE = 0.020, *z* = −4.97, ratio = 0.91, Holm-adjusted *p* < 0.001. Responses were also faster following feature-sharing distractors than following non-feature-sharing distractors, *b* = −0.069, SE = 0.020, *z* = −3.46, ratio = 0.93, Holm-adjusted *p* = 0.001. The difference between the non-feature-sharing and no-distractor conditions was not significant, *b* = −0.030, SE = 0.020, *z* = −1.50, ratio = 0.97, Holm-adjusted *p* = 0.135.

In the four-cue condition, non-feature-sharing distractors produced slower responses than the no-distractor condition, *b* = 0.074, SE = 0.020, *z* = 3.71, ratio = 1.08, Holm-adjusted *p* < 0.001. Responses were also slower following non-feature-sharing distractors than following feature-sharing distractors, *b* = 0.060, SE = 0.020, *z* = 2.98, ratio = 1.06, Holm-adjusted *p* = 0.006. The feature-sharing and no-distractor conditions did not differ significantly, *b* = 0.014, SE = 0.020, *z* = 0.71, ratio = 1.01, Holm-adjusted *p* = 0.475.

Overall, reaction times were fastest when attention was narrowly focused and generally became slower as attention was distributed across more retro-cued items. Distractor effects varied across cue conditions: no reliable effects were found in the no-cue condition; non-feature-sharing distractors selectively slowed responses in the one-cue and four-cue conditions; both distractor types slowed responses in the two-cue condition; and feature-sharing distractors facilitated responses in the three-cue condition.

### 3.3 Discussion

Experiment 2 extended the Experiment 1 findings from external attention during encoding to internal attention during maintenance. The main effect of retro-cue load showed that accuracy was highest and responses were fastest when only a small number of items was prioritized, whereas performance declined as attention was distributed across more maintained representations. This pattern again indicates that internal attentional prioritization was graded and capacity limited rather than all-or-none. Retro-cues were most effective when they concentrated priority on a small amount of items, supporting the view that internal attention increases the accessibility of selected memory representations (Griffin and Nobre, 2003; Myers et al., 2017; Souza and Oberauer, 2016). The convergent decrease in accuracy and increase in reaction time as more items were retro-cued indicate that internal prioritization remains resource limited and is constrained by competition for access among maintained representations (Oberauer, 2019; Rerko et al., 2014).

The interaction between retro-cue load and distraction further showed that the consequences of irrelevant input depended on how broadly internal attention was distributed. When attention was narrowly focused, distractors produced little reliable impairment, suggesting that strongly prioritized representations were relatively resistant to interference. By contrast, in the four-cue and no-cue conditions, non-feature-sharing distractors reduced accuracy relative to the no-distractor condition. Feature-sharing distractors did not differ from the no-distractor baseline and, in the no-cue condition, produced higher accuracy than non-feature-sharing distractors. Because the feature-sharing condition did not exceed the no-distractor baseline, this pattern should be interpreted as reduced interference relative to unrelated distraction rather than genuine memory facilitation. One possible explanation is that broadly distributed or absent prioritization left several relatively weak memory traces vulnerable to novel, mismatching input, whereas a distractor sharing a feature with the memory array introduced less discrepant evidence.

The reaction-time effects provided additional evidence. Both distractor types slowed responses in the two-cue condition, with a larger cost for non-feature-sharing distractors, whereas feature-sharing distractors accelerated responses in the three-cue condition without improving accuracy. Because these effects were confined to reaction time, they are more appropriately interpreted as changes in retrieval, decision efficiency, or response preparation than as changes in memory fidelity. The three-cue facilitation may partly reflect temporal alerting, as the distractor onset could have served as a warning signal before the probe (van Ede et al., 2017, 2018). Overall, Experiment 2 shows that internal attention protects VWM most effectively when priority is concentrated on a small number of representations; as prioritization broadens, performance declines and susceptibility to distraction becomes increasingly dependent on distractor similarity.

## 4 General discussion

The present study examined how attentional prioritization and distractor similarity jointly determine the robustness of VWM. Across two experiments, performance was best when a small number of items was prioritized and declined when attention was distributed across more items or when no cue was provided. The convergence of accuracy and reaction time for this cue-load effect supports resource-based and priority-based accounts of VWM, according to which memory performance depends on how limited attentional and representational resources are allocated across competing items (Bays and Husain, 2008; Cowan, 2011; Luck and Vogel, 2013; Oberauer, 2019).

The central theoretical contribution is that feature-sharing distractors were not uniformly more harmful than non-feature-sharing distractors. Under broad or absent attentional guidance, feature-sharing distractors were often less disruptive; however, they did not improve accuracy beyond the no-distractor baseline. The findings therefore challenge a simple monotonic account in which greater feature overlap necessarily produces greater interference, but they do not demonstrate mnemonic facilitation. The attentional priority and VWM resource accounts explain the cue-load effect by proposing that per-item accessibility and precision decrease as more items are prioritized. Interference and signal-intrusion accounts explain the conditional distractor effects by proposing that behavioral impact depends on the similarity structure of the memory set, which signals remain accessible, and how distractor-derived evidence enters retrieval and decision processes (Lin and Oberauer, 2022; Oberauer and Lin, 2017; Zhang and Lewis-Peacock, 2025).

These accounts suggest a two-stage interpretation. First, attentional prioritization determines the accessibility and precision of the relevant representations. Second, distractor input interacts with this representational state. When one item is strongly prioritized, distractor type has little effect. When attention is broadly distributed, non-feature-sharing input may produce greater novelty or mismatch and more disruptive signal intrusion, whereas feature-sharing input is relatively less disruptive. When multiple representations remain accessible, overlap may also increase ambiguity, but the present evidence for this possibility was confined largely to reaction time and should remain exploratory. This interpretation is consistent with evidence that distraction can produce attraction biases, swap-like errors, precision changes, and guessing rather than a single unitary loss (Lorenc et al., 2021; Zhang and Lewis-Peacock, 2023a,b, 2025) and with interference models locating retrieval competition in a similarity space (Lin and Oberauer, 2022; Oberauer and Lin, 2017).

The comparison between pre-cues and retro-cues further clarifies the relationship between external and internal attention. Pre-cues influenced which items were efficiently encoded into VWM, whereas retro-cues altered the accessibility of items already stored. Both experiments showed that focused prioritization reduced vulnerability to distraction and that feature similarity exerted a stronger influence when prioritization was broad or absent. However, a direct quantitative comparison is limited by the use of separate participant samples and different retention timelines. In Experiment 1, the probe appeared 1,000 ms after memory-array offset; in Experiment 2, it appeared 1,850 ms after memory-array offset because the retro-cue and additional delays were inserted during maintenance. The longer interval in Experiment 2 could increase decay or interference and is therefore a potential confound for between-experiment differences in effect magnitude. Because the hypotheses and Cue Type × Distractor Type interactions were tested within each experiment, and no formal Experiment × Cue Type × Distractor Type model was fitted, this timing difference does not invalidate the within-experiment conclusions. It does, however, prevent attributing any cross-experiment difference solely to external vs. internal attention. A future within-subject experiment should equate retention duration and cue-probe timing.

Accuracy and reaction time provided converging evidence for the cue-load effect: broader prioritization reduced accuracy and slowed responses. Distractor contrasts were less convergent. Accuracy is the primary measure of whether memory supported correct recognition, whereas reaction time was computed only on correct trials and can also be affected by response threshold, temporal alerting, and motor preparation. Accordingly, a distractor effect accompanied by an accuracy change was interpreted as evidence of altered memory performance, whereas reaction time only effects were described as changes in retrieval or decision efficiency and treated as exploratory. This distinction avoids inferring memory competition from faster or slower responses alone.

Several limitations should be acknowledged. First, the present study used behavioral measures only. Although the results are consistent with reduced mismatch or signal-intrusion accounts, they cannot determine whether feature-sharing distractors altered maintained representations, retrieval evidence, or decision processes. Eye tracking, EEG, or neural decoding methods could help distinguish these possibilities (Gresch et al., 2024; Nobre and Gresch, 2025). Second, feature-sharing distractors always matched an unprobed memory item, and color-sharing and shape-sharing trials were collapsed. The design therefore cannot distinguish array-context consistency, feature-specific intrusion, binding confusion, or novelty-based effects. Future work should manipulate target relevance and feature dimension factorially and use continuous-report measures that reveal the distribution of errors. Third, the different maintenance durations and separate samples limit direct comparisons between experiments. Fourth, the apparent advantage of feature-sharing distractors should be interpreted as reduced interference relative to unrelated distractors, not as evidence that distraction improves memory beyond a distraction-free baseline.

Overall, the findings support a dynamic account of VWM in which the impact of distraction is determined jointly by top-down attentional prioritization and bottom-up feature overlap. Feature similarity does not have a fixed effect, but the present evidence supports conditional relative disruption rather than mnemonic support. The effect depends on the attentional state and decision context, and the reaction time only patterns require confirmation with designs that separate memory, temporal alerting, and response processes.

## 5 Conclusion

The present study demonstrates that both external and internal attention enhance VWM by prioritizing task-relevant representations during encoding and maintenance. These benefits are strongest when only a small number of items are prioritized and decline as attentional selection becomes more broadly distributed. Crucially, feature-sharing distractors were sometimes less disruptive than non-feature-sharing distractors when prioritization was weak or diffuse, but they did not improve accuracy relative to the no-distractor baseline. Accuracy and reaction time converged on cue-load effects but dissociated for several distractor contrasts. These findings support a dynamic, resource-limited account in which the behavioral consequence of sensory similarity depends on attentional allocation and representational accessibility, while leaving open whether the observed relative sparing arises from reduced novelty, altered signal intrusion, or decision-level processes.

## Data Availability

The datasets presented in this study can be found in online repositories. The names of the repository/repositories and accession number(s) can be found at: https://osf.io/3q5pa/files/osfstorage.
